# Treatment guesses in the Treatment for Adolescents with Depression Study: Accuracy, unblinding and influence on outcomes

**DOI:** 10.1177/00048674231218623

**Published:** 2023-12-21

**Authors:** Jon Jureidini, Joanna Moncrieff, Julie Klau, Natalie Aboustate, Melissa Raven

**Affiliations:** 1 Critical and Ethical Mental Health Research Group, Robinson Research Institute, The University of Adelaide, Adelaide, SA, Australia; 2 Division of Psychiatry, Division of Psychiatry, University College London, London, UK

**Keywords:** Randomised controlled trial blinding, antidepressant, selective serotonin reuptake inhibitor, placebo, adolescent depression

## Abstract

**Objective:**

We evaluated the presence and impact of unblinding during the influential Treatment for Adolescents with Depression Study (ClinicalTrials.gov Identifier: NCT00006286).

**Method:**

Our analysis was part of a Restoring Invisible and Abandoned Trials reanalysis. Treatment for Adolescents with Depression Study trialled fluoxetine, placebo, cognitive behaviour therapy or their combination, in treating adolescents with major depressive disorder. We analysed the accuracy of guesses of fluoxetine or placebo allocation, and their effects on change in Children’s Depression Rating Scale-Revised at 12 weeks.

**Results:**

Of 221 participants allocated to fluoxetine or placebo, 151 adolescents (68%) had their guess about pill-treatment-arm allocation recorded at week 6, and guesses were recorded for 154 independent evaluators, 159 parents and 164 pharmacotherapists. All of these groups guessed treatment allocation more accurately than would be expected by chance (60–66% accuracy; all *p*-values ⩽ 0.004). Guesses did not become more accurate between 6 and 12 weeks and were not predicted by adverse events, though event documentation was poor. Treatment guess had a substantial and statistically significant effect on outcome (Children’s Depression Rating Scale-Revised change mean difference 9.12 [4.69; 13.55], β = 0.334, *p* < 0.001), but actual treatment arm did not (1.53 [−2.83; 5.89], β = 0.056, *p* = 0.489). Removing guess from the analysis increased the apparent effect of treatment arm, making it almost statistically significant at the conventional alpha-level of 0.05 (*p* = 0.06).

**Conclusions:**

For Treatment for Adolescents with Depression Study, treatment guesses strongly predicted outcomes and may have led to the exaggeration of drug effectiveness in the absence of actual effects. The integrity of double-blinding in trials should be routinely assessed and reported.

## Introduction

There has been a significant rise in the use of antidepressants throughout the world in recent decades, with dramatic increases among children and teenagers (Jack et al., 2020), despite concerns about a heightened risk of suicidality (Hengartner, 2020), the influence of financial interests (Hengartner, 2020) and general concern about the use of drugs in children (Klau et al., 2021). Use of antidepressants is based on placebo-controlled trials that indicate modest differences between antidepressants and placebo (Cipriani et al., 2018). In children and adolescents only, fluoxetine is thought to have a possibly meaningful benefit over placebo (Zhou et al., 2020).

A recurrent concern about antidepressant trials is the possibility of unblinding, which may lead to amplified expectancy or placebo effects in people allocated to active drugs, and reduced placebo effects in those taking placebo, thereby exaggerating the effects of treatment (Even et al., 2000). Placebo effects are likely to be especially relevant in a condition like depression, where lack of hope and optimism are part of the problem itself (Kirsch et al., 2002). Despite this, the success of blinding is rarely tested (Scott et al., 2022). Two recent systematic reviews found only seven and nine trials which tested and reported the integrity of blinding (Lin et al., 2022; Scott et al., 2022). One concluded there was no overall evidence of unblinding in a combined analysis (Lin et al., 2022), but both reviews found evidence of unblinding in some trials.

It is usually assumed that unblinding is caused by people recognising whether or not they are in a medicated state due to the occurrence of side effects or other, subtle physical and mental alterations produced by drugs (Rief and Glombiewski, 2012). However, some authors point out that unblinding may occur because of therapeutic effects. There is currently little evidence that could clarify this issue, though several studies across diverse conditions show that whether people guess they are taking active drug or placebo predicts clinical improvement independently of the effects of the drug (Bingel et al., 2011; Chen et al., 2011). Clinicians’ guesses can also independently predict outcome (Chen et al., 2015). These studies imply that unblinding could influence the results of randomised trials, but further evidence is needed to explore this possibility.

During a reanalysis of the Treatment for Adolescents with Depression Study (TADS), we gained access to a dataset that gave insight into the extent of unblinding and the effects of treatment guesses on outcomes. Funded by the National Institute of Mental Health (NIMH) and conducted by the Duke Clinical Research Institute, TADS was a highly influential multicentre randomised controlled trial (RCT) *examining the comparative effectiveness of established treatments for adolescents with major depressive disorder (MDD)* (National Institute of Mental Health, 2000: 6). It was originally reported as showing beneficial effects of fluoxetine (March et al., 2004), and is frequently cited as supporting the use of antidepressants in children and adolescents (e.g. Murphy et al., 2021). The four treatment arms were fluoxetine only, cognitive-behavioural therapy (CBT) only, fluoxetine–CBT combination treatment and placebo only. TADS was effectively two studies – a blinded comparison of fluoxetine and placebo, and open administration of CBT with and without fluoxetine (Jureidini et al., 2004). The methodology was reported by the TADS Team (2003), and the main efficacy results were published by March et al. (2007).

The trial involved adolescents, their parents, blinded independent evaluators (IEs) and pharmacotherapists (who conducted all the pill-related visits, including dispensing, adverse event (AE) reporting and patient symptom questionnaires, but were blind to medication status during Stage 1) making repeated guesses about treatment allocation. All were blinded with respect to fluoxetine and placebo. The overall aim of the current study was to examine the accuracy with which adolescents and other participants guessed treatment allocation (a measure of unblinding) and to what extent guesses influenced outcomes. We also explored whether actual treatment allocation (treatment arm) had an independent effect from that of adolescents’ guesses.

## Method

The original TADS (ClinicalTrials.gov Identifier: NCT00006286) was a Phase III multicentre, parallel four-arm randomised controlled superiority trial of fluoxetine (National Library of Medicine (US), 2014), conducted at 13 sites across the United States during 2003, involving 439 adolescents aged 12–17 years, who met the *Diagnostic and Statistical Manual of Mental Disorders* (4th ed.; DSM-IV) criteria for MDD (full details are described in TADS documents and previous publications [National Institute of Mental Health, 2000; TADS Team, 2003]).

Our overall TADS reanalysis is being was conducted as part of the Restoring Invisible and Abandoned Trials (RIAT) initiative established in 2013 to enhance accountability in clinical trials (Doshi et al., 2013), with data obtained from the National Database for Autism Research (NDAR, https://nda.nih.gov/about.html; Collection ID#2145).

Two primary outcome variables were specified in the study protocol: *Change in IE-administered CDRS-R total score across 12 and 36 weeks of treatment* (National Institute of Mental Health, 2000: 17) and response rate on a clinician-rated global impression scale. Our analysis used the Children’s Depression Rating Scale–Revised (CDRS-R), a 17-item clinician-rated scale assessing depressive symptoms experienced in the preceding week using a semi-structured interview (Poznanski and Mokros, 1996). The original TADS team applied a variety of dated imputation rules to each CDRS-R item and their dataset provided both imputed and unimputed scores. We used unimputed scores in our analyses. Our analysis of the accuracy of guesses and their impact on outcomes focuses on the first 12 weeks of treatment, since guesses were obtained only at weeks 6 and 12, and because this was the period of randomised treatment.

**Figure 1.**
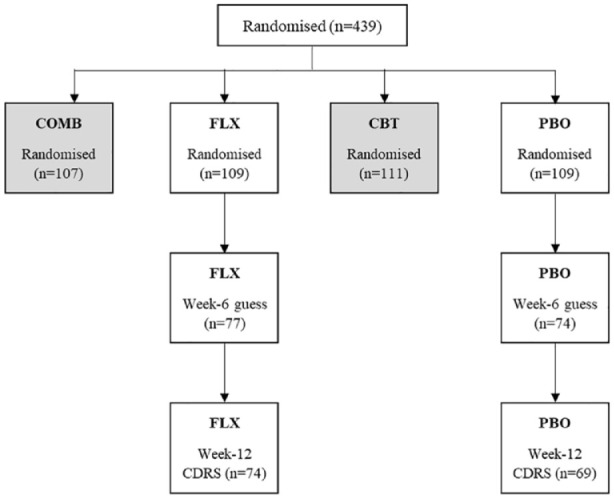
Randomised treatment allocations in TADS showing adolescents for whom guesses were documented at week 6 and who had CDRS-R scores at week 12.

For adolescents, parents and pharmacotherapists (the clinician’s dispensing pills), only data from the blinded comparison of fluoxetine (*n* = 109) and placebo (*n* = 111) is included in our evaluation (see Figure 1). For IEs, who were blinded to all four treatment allocations, a larger dataset comprising guesses from all four treatment arms was available (*n* = 390). For analysis of guess accuracy, we combined IE guesses into two categories: guessed fluoxetine or combination treatment; or guessed not fluoxetine (i.e. either placebo or CBT treatment). Our analysis of the impact of guesses on outcomes only included fluoxetine and placebo treatment arms.

For week-6 adolescent guesses (*n* = 151), there were no baseline differences between those who guessed fluoxetine and those who guessed placebo, for gender, site, race, age, treatment expectancy or severity of depression (Table 1). There was also no difference in certainty of week-6 guesses for those guessing fluoxetine versus placebo, nor in treatment expectancy between those who guessed fluoxetine and those who guessed placebo (though our analysis lacked power to detect a difference).

**Table 1.** Baseline characteristics of adolescents who guessed fluoxetine or placebo at week 6.

	Total (*n*, %)	Guessed fluoxetine (%)	Guessed placebo (%)	*p*-value
Gender	151 (43.7% male)	44.8% male	42.9% male	0.813^a^
Race	151 (72.9% Caucasian)	74.6% Caucasian	71.4% Caucasian	0.487^b^
Site (*n* = 13)	151			0.108^b^
Treatment expectancy^c^	139 (51.8% much/very much improved)	54% much/very much improved	50% much/very much improved	0.641^a^
Confidence in guesses at week 6	149 (54.4% high)	61.2% high	48.8% high	0.130^a^
Mean (SD) CDRS-R at baseline	151	61.2 (10.4)	59.5 (11.2)	0.344^d^
Mean (SD) age at baseline	151	14.5 (1.4)	14.5 (1.7)	0.968^d^

SD: standard deviation; CDRS-R: Children’s Depression Rating Scale–Revised.

a Chi square.

b Analysed using Stata tabchi command, reported Likelihood ratio *p*-value.

c Responses were scored on a Likert-type scale ranging from 1 (very much improved) to 7 (very much worse). To avoid problems with small cell numbers, we collapsed treatment expectancy into a two-category variable, one including *much improved* or *very much improved* and the other including the other five categories encompassing minimal or no improvement or worsening.

d *t*-test, assuming equal variances.

The original trial received ethical approval from the Duke University Medical Center and institutional review boards at each recruiting site. As our reanalysis used secondary, de-identified data we received an exemption from ethical approval (University of Adelaide Human Research Ethics Committee reference 33958). The original TADS team stated they received written informed consent from all trial participants and their guardians (TADS Team, 2003).

## Blinding

Treatment blindness for parents, adolescents and IEs was assessed at weeks 6 and 12 by asking them to guess which treatment the adolescent was receiving and rate their level of certainty (National Institute of Mental Health, 2000: 91). Pharmacotherapists recorded guesses on multiple occasions throughout the 12 weeks, guessing an average of 5.4 times for each patient. To effectively compare with guesses by other participants, we generated a variable for the pharmacotherapist with three time-points: baseline, week 6 and week 12. Any guess recorded from 0 to 7 days was classified as baseline; guesses between 5 and 7 weeks were classified as week 6, and between 11 and 13 weeks as week 12. Where there was more than one record within any of these time spans, the closest guess to each time-point was chosen.

## Accuracy of guesses

Percentage accuracy of guesses was calculated for all four guessing groups, i.e. adolescents, IEs, parents and pharmacotherapists. We decided not to use a blinding index (Bang et al., 2004; James et al., 1996), both because of difficulties of interpretation (see, for example, Bechara et al., 1997) and because blinding indexes assume there is an *I don’t know* category, which was not the case for TADS.

As well as comparing the proportion of accurate guesses at weeks 6 and 12, we examined the sub-sample for whom guesses were available at both time-points and analysed how guesses changed over time. We also checked for any effect of guess certainty, collapsing a five-point Likert-type scale to a dichotomous low or neutral (1–3) or high (4–5) certainty.

We explored whether correct guessing was associated with total AEs experienced. To eliminate masking of signal by noise from events unlikely to be related to medication, we repeated the analysis restricted to effects listed in the TADS protocol as associated with fluoxetine (*expected AEs*). We also examined the *physical symptoms checklist* (National Institute of Mental Health, 2000: 90), a self-report scale filled out by the adolescents every 6 weeks, to see if the number of *health problems* experienced was correlated with guesses.

## Association between guesses and outcomes

We selected the CDRS-R as our primary outcome for the present analysis because it is a continuous variable, facilitating more robust modelling and it is the one most often cited in support of the effectiveness of fluoxetine in TADS. First, we compared the outcome between the sub-sample of 151 participants for whom guesses were recorded and the complete sample (*n* = 221) allocated to fluoxetine or placebo, to ensure that the sub-sample was comparable to the total pill-only sample. Then we analysed mean change in outcome from baseline to week 12 according to treatment arm and guess category at week 6 (i.e. allocated to FLX, guessed FLX; allocated to FLX, guessed PBO; allocated to PBO, guessed PBO; and allocated to PBO, guessed PBO) using univariate tests. We selected guesses at week 6 because these were considered less likely to be influenced by therapeutic effects than those at week 12. We also carried out a sensitivity analysis of the impact of certainty of guesses on those comparisons. Finally, we conducted multiple linear regression to examine the effect of adolescent guesses at week 6 on week 12 outcome.

The following models were examined:

Model 1: CDRS-R week-12 scores were regressed on adolescent guesses at week 6, with baseline CDRS-R, age at baseline and treatment expectancy (a seven-category variable which we dichotomised to avoid small cell numbers), which was hypothesised to predict outcome, as predictors.
Model 2: repeated Model 1 and included treatment arm as a predictor.
Model 3: CDRS-R week-12 scores were regressed on treatment allocation with baseline CDRS-R, age at baseline and dichotomised treatment expectancy as predictors (omitting the *guess* variable).

Analyses were performed using Stata version 16 (StataCorp, Texas, USA).

In addition to statistical measures, we also considered the clinical significance of the findings (Moncrieff and Kirsch, 2015).

## Results

### Baseline characteristics

In all, 221 adolescents were allocated fluoxetine or placebo (pill-only). Adolescents, parents, pharmacotherapists and IEs’ guesses were available for 65–75% of pill-only participants at weeks 6 and 12. Participants for whom a guess was recorded at week 6 and who had a CDRS-R rating at week 12 were modelled in our primary reanalysis. Figure 1 shows the attrition for adolescent guesses.

Similarly, there were no meaningful differences in the baseline characteristics of adolescents for whom there were guesses by IEs, parents and pharmacotherapists (Tables S1, S3 and S6).

### CDRS-R outcomes in sub-sample with guesses compared with outcomes in total pill-only sample

Guesses of 151 adolescents and 154 IEs were recorded at week 6. In both these guessing groups, change in CDRS-R over 12 weeks was similar to change for the total sample allocated to pill-only (Figure S1), suggesting that approximately 70% of adolescents for whom guesses were recorded constituted a representative sub-sample.

### Accuracy of guesses

For all guessers, the percentage of correct guesses was similar for weeks 6 and 12, and all four groups guessed treatment allocation more accurately than the 50% (25% for IEs) that would be expected by chance at both time-points:

- 62% of adolescents guessed accurately at week 6 (χ^2^_1_ = 8.38, *p* *=* 0.004) and 64% at week 12 (χ^2^_1_ = 11.59, *p* *=* 0.001);
- 60.4% of parents guessed accurately at week 6 (χ^2^_1_ = 6.87, *p* = 0.009) and 64.1% at week 12 (χ^2^_1_ = 11.67, *p* = 0.001).

IEs were blind to all four treatment allocations and therefore had more guesses (352 at week 6 and 353 at week 12); 60.0% of guesses were correct at week 6 (χ^2^_1_ = 14.74, *p* *<* 0.001) and 63.1% at week 12 (χ^2^_1_ = 25.36, *p* *<* 0.001).

Pharmacotherapist guesses at baseline were not significantly different from chance (χ^2^_1_ = 0.26, *p* = 0.610), guessing only 52.9% correctly. However, at week 6, 62.2% guessed accurately (χ^2^_1_ = 10.22, *p* = 0.001) and 65.8% guessed accurately at week 12 (χ^2^_1_ = 11.92, *p* = 0.001).

#### Change in guess

We examined whether guesses changed between weeks 6 and 12, to see if participants’ ability to identify their allocated treatment became more accurate over the duration of treatment. Guesses were recorded for 123 adolescents at both weeks 6 and 12. The majority (*n* = 95, 77.2%) did not change their guess, 3 (2.4%) changed from an incorrect to a correct guess and 25 (20.3%) changed from a correct to an incorrect guess.

#### Impact of adverse effects

Exploring whether the experience of adverse effects predicted correct guessing, we found no meaningful association between accuracy of guess and reported AEs (see summaries in Supplementary Tables S14–17).

### Impact of guesses on outcome

**Figure 2.**
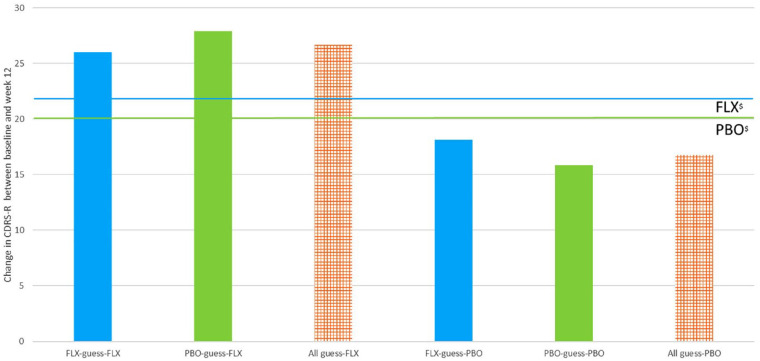
Change from baseline to week 12 in CDRS-R according to adolescents’ guesses about treatment allocation at week 6. ^$^The upper line represents the mean change in CDRS-R for adolescents allocated to FLX and the lower line represents the change in CDRS-R for adolescents allocated to PBO.

Figure 2 shows that those who guessed they were taking fluoxetine at week 6 (*n* = 67), regardless of whether they were correct, had greater change in CDRS-R than those who were actually allocated to fluoxetine (*n* = 77; blue bars), and also greater change than those who guessed they were taking placebo. In fact, adolescents who guessed they were on fluoxetine, but were actually allocated to placebo, demonstrated the largest improvement in CDRS-R. Conversely, those who guessed placebo correctly or incorrectly (*n* = 84; Figure 2, right-hand columns) fared worse than those who were actually allocated to placebo (*n* = 69; Figure 2, green line), and worse than those who guessed they were taking fluoxetine. Overall mean change among those who guessed they were taking fluoxetine at −26.98 (SD 16.19) was 10 points higher than the mean change of −16.65 (SD 14.73) for those who guessed they were taking placebo, a statistically significant difference, *t*(139) = −3.96, Cohen’s *d* = 0.67, *p* < 0.001. By comparison, the overall mean change in CDRS-R over 12 weeks for those actually allocated to fluoxetine (*n* = 72) was −22.49 (SD 16.62) and −19.84 (SD 15.71) for those allocated to placebo (*n* = 69), a clinically non-significant difference, *t*(139) = −0.97, Cohen’s *d* = 0.16, *p* = 0.33.

For IEs, the difference in mean change was −22.92 (SD 15.75) for participants guessed to be on fluoxetine, and −14.49 (SD 16.63) for those guessed to be on placebo, *t*(140) = −2.84, Cohen’s *d* = 0.53, *p* = 0.005. A similar pattern was seen with parent and pharmacotherapist guesses (Table S1).

### Impact of certainty of guesses on outcome

**Figure 3.**
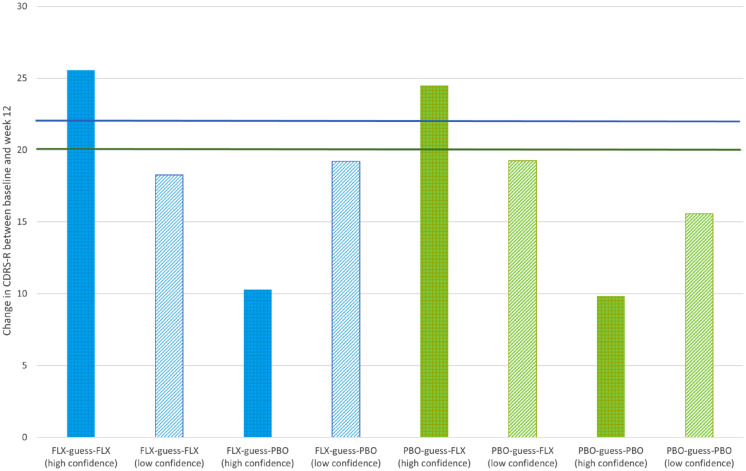
Change from baseline to week 12 in CDRS-R, according to adolescents’ guesses and confidence of guesses about treatment allocation at week 6. ^$^The upper line represents the mean change in CDRS-R for adolescents allocated to FLX, and the lower line represents the mean change for adolescents allocated to PBO.

Participants’ confidence ratings about their guesses on the dichotomised Likert-type scale were high for 81 adolescents and low for 68. Figure 3 shows that higher confidence in adolescent’s guess about treatment allocation increased the concordance between guesses and outcome.

### Linear regression of change in CDRS-R

#### Adolescents

The overall regression for Model 1 (CDRS-R week-12 scores regressed on adolescent guesses at week 6, with baseline CDRS-R, age at baseline and dichotomised treatment expectancy as predictors) was statistically significant, *R*^2^ = 0.185, *F*(4, 125) = 5.92, *p* < 0.001, adjusted *R*^2^ = 0.159; Table 2. The mean week-12 CDRS-R score for those who guessed fluoxetine was 33.93 and 43.50 for those who guessed placebo. The difference of 9.59 (95% CI = [5.23, 13.95]) was statistically significant (β = 0.351, *p* < 0.001), and reaches the threshold of clinical significance (Moncrieff and Kirsch, 2015). Adolescent guesses at week 6 explained 12.3% of the variance in the outcome variable at week 12.

**Table 2.** Regression model 1: adolescent treatment guess at week 6 as predictor of CDRS-R at week 12 (*R*^2^ = 0.185[*N* = 130, *p* < 0.001], adjusted *R*^2^ = 0.159).

Variable	B	Robust SE	95% CI	β	*t*	*p*	ω^2^
Guess at week 6	9.59	2.20	[5.23, 13.95]	0.351	4.35	<0.001	0.12
Treatment expectancy	3.31	2.16	[−0.97, 7.59]	0.122	1.53	0.129	0.01
CDRS-R baseline	0.19	0.10	[−0.00, 0.38]	0.151	1.94	0.055	0.02
Age at baseline	1.30	0.64	[0.03, 2.58]	0.151	2.03	0.045	0.02

CDRS-R: Children’s Depression Rating Scale–Revised; SE: standard error; CI: confidence interval.

Model 2 found that including allocated treatment arm as an additional predictor did not noticeably change the results obtained in Model 1 (Table 3). The regression was also significant, *R*^2^ = 0.188, *F*(5, 124) = 4.68, *p* < 0.001, adjusted *R*^2^ = 0.155, but allocated treatment did not have any independent effect on outcome (β = 0.056, *p* = 0.489). Guess at week 6 remained a statistically significant predictor of outcome at week 12 (β = 0.334, *p* < 0.001), explaining 10.2% of the variance in the outcome. Treatment allocation contributed close to no impact on the outcome.

**Table 3.** Regression model 2: adolescent treatment guess at week 6 as predictor of CDRS-R at week 12, including treatment allocation as a predictor (*R*^2^ = 0.188 [*N* = 130, *p* < 0.001], adjusted *R*^2^ = 0.155).

Variable	B	Robust SE	95% CI	β	*t*	*p*	ω^2^
Guess at week 6	9.12	2.24	[4.69, 13.55]	0.334	4.07	**<0.001**	0.10
Treatment expectancy	3.49	2.13	[−0.72, 7.71]	0.128	1.64	0.104	0.01
CDRS-R baseline	0.18	0.10	[−0.01, 0.37]	0.144	1.85	0.067	0.02
Age at baseline	1.31	0.64	[0.04, 2.59]	0.152	2.04	**0.044**	0.02
Treatment allocation	1.53	2.20	[−2.83, 5.89]	0.056	0.69	0.489	−0.01

CDRS-R: Children’s Depression Rating Scale–Revised; SE: standard error; CI: confidence interval.

Model 3, which explored the role of treatment allocation without including guess, was statistically significant, *R*^2^ = 0.088, *F*(4, 125) = 2.76, *p* = 0.031, adjusted *R*^2^ = 0.059; Table 4. It showed a trend effect for actual treatment allocation (β = 0.160, *p* = 0.060) and a statistically significant effect of age at baseline (β = 0.169, *p* = 0.038, ω^2^ = 0.022).

**Table 4.** Regression model 3: treatment allocation as a predictor of CDRS-R at week 12, including baseline CDRS-R, age at baseline and treatment expectancy as predictors (*R*^2^ = 0.088 [*N* = 130, *p* = 0.031], adjusted *R*^2^ = 0.059).

Variable	B	Robust SE	95% CI	β	*t*	*p*	ω^2^
Treatment allocation	4.34	2.29	[−0.19, 8.87]	0.16	1.90	0.060	0.02
CDRS-R baseline	0.12	0.11	[−0.07, 0.35]	0.11	1.28	0.203	0.01
Age at baseline	1.46	0.69	[0.08, 2.83]	0.17	2.10	**0.038**	0.02
Treatment expectancy	4.02	2.26	[−0.46, 8.49]	0.15	1.78	0.078	0.02

CDRS-R: Children’s Depression Rating Scale–Revised; SE: standard error; CI: confidence interval.

### IEs

The regression for IEs was restricted to those 154 adolescents allocated to FLX or PBO, for whom an IE guess was recorded at week 6. Again, guess at week 6 was a statistically significant predictor of outcome (β = 0.224, *p* = 0.012), but treatment allocation was not (Table 5).

**Table 5.** Regression model 2: IE guess at week 6 as predictor of CDRS-R at week 12, including treatment allocation as a predictor (*R*^2^ = 0.125 [*N* = 142, *p* = 0.001], adjusted *R*^2^ = 0.099).

Variable	B	Robust SE	95% CI	β	*t*	*p*	ω^2^
Guess at week 6	6.81	2.69	[1.50, 12.13]	0.224	2.54	**0.012**	0.046
CDRS-R baseline	0.11	0.11	[−0.10, 0.32]	0.088	1.07	0.284	0.001
Age at baseline	1.55	0.67	[0.22, 2.88]	0.180	2.31	**0.023**	0.027
Treatment allocation	3.16	2.26	[−1.31, 7.63]	0.115	1.40	0.164	0.007

CDRS-R: Children’s Depression Rating Scale–Revised; SE: standard error; CI: confidence interval.

Similar results were seen for all models of 6-week guesses by parents and pharmacotherapists, and for 12-week guesses for all four guessing groups (not shown; see Supplementary Materials), indicating a high level of consistency.

## Discussion

### Principal findings

Adolescents, IEs, parents and pharmacotherapists all guessed treatment allocation more accurately than would be expected by chance. A few of those who guessed at both weeks 6 and 12 changed their guesses and guesses did not become more accurate with time. Our analysis suggests that people can guess the nature of their pills independently of therapeutic effects, since these were not apparent in this study. We did not confirm whether adverse effects revealed the identity of medication, but these were poorly recorded and unblinding may be attributable to more subtle mental and physical alterations produced by drugs. We also showed that unblinding can inflate the apparent effects of drugs in randomised trials.

TADS is commonly perceived as having demonstrated the effectiveness of fluoxetine, because the original publication highlighted the superiority of combination treatment with fluoxetine and CBT over placebo (March et al., 2004). However, the original analysis (March et al., 2004) and our overall reanalysis (manuscript in preparation), showed no statistically or clinically significant difference between the fluoxetine and placebo arms on the CDRS-R. Thus drug-related therapeutic effects cannot account for the accuracy of guesses, as has been suggested in the past.

We expected that the presence or absence of AEs would be a contributor to unblinding. Our analysis was not consistent with this hypothesis, with the presence of AEs having no apparently meaningful influence on guessing; however, adverse effects were reported by the TADS Team to have been inadequately documented (Emslie et al., 2006). Moreover, it may be that adolescents and other guessers detected more subtle effects from fluoxetine that alerted them to treatment allocation, but were not of sufficient weight to be reported or classified as AEs. Fluoxetine may have a relatively benign short-term side-effect profile in children and adolescents as well as adults (Zhou et al., 2020). This suggests that guesses about other antidepressants, such as paroxetine or sertraline, might be more accurate.

Adolescents, parents, pharmacotherapists and IEs’ guesses all predicted outcomes with substantial effects (apart from pharmacotherapists’ guess at baseline when medication or placebo had not been initiated). The differences in mean 12-week CDRS-R change scores between participants and IEs who guessed fluoxetine and those who guessed placebo at week 6 were 16.6 points for adolescents and 8.4 for IEs, which considerably exceed the difference between fluoxetine and placebo in the overall TADS (3.23 at week 12 in March et al. (2004)). The differences exceed thresholds of clinical significance (Moncrieff and Kirsch, 2015) and equate to substantial effect sizes of 0.67 and 0.53, respectively, which are more than twice the effect sizes reported in an influential meta-analysis of placebo-controlled trials of antidepressants (Cipriani et al., 2018). Our findings are consistent with the large effects demonstrated in the experimental study of expectancy manipulation in people treated with escitalopram for social anxiety disorder (Faria et al., 2017).

Our results are also consistent with other trials of antidepressants that show that participants’ beliefs about the treatment they receive are strongly associated with outcomes, independently of the effect of treatment (Chen et al., 2015; Faria et al., 2017; Laferton et al., 2018). Some studies have also found that physician guesses predict outcomes (Chen et al., 2015; Laferton et al., 2018), consistent with our data from pharmacotherapists. The effects of guesses on outcome is likely to reflect implicit and explicit messages about the benefits of medication that are presented in advertising and popular culture (Hengartner, 2022).

Although treatment arm had no statistically significant effect on outcome, independent of guess, when guess was removed from the analysis, treatment arm had a marginal effect. This demonstrates that unblinding can influence the results of a randomised trial independently from possible therapeutic effects. More accurate guesses, as might occur with a drug with stronger side effects, might spuriously inflate treatment effects further.

### Strengths and weaknesses

This is one of few studies to report the effect of guessing by patients and others on study outcomes (Scott et al., 2022). However, it is based on an incomplete sub-sample of the TADS – although guesses were recorded for approximately 70% of adolescents, only 56% of pill-only adolescents had them recorded at both weeks 6 and 12.

Participants were not asked to guess until week 6 of the trial; it would have been useful to know the accuracy and influence of earlier guesses because of the suggestion that therapeutic effects contribute to unblinding. The lack of difference between fluoxetine and placebo in the current study and the lack of change between weeks 6 and 12 reduce the likelihood that unblinding was attributable to therapeutic effects.

We were not able to use a blinding index because they rely on there being *don’t know* guesses and therefore we were not able to summarise blinding integrity in one or two figures as these indexes facilitate. There is debate about the best sort of blinding index, however, and since they combine important data, the recommendation is still to present all the data on guesses in different groups as we have done (Kolahi et al., 2009).

Some previous studies have found that unblinding correlates with side effects (Chen et al., 2015) but some do not (Laferton et al., 2018) and there remain questions about the adequacy of side-effect recording (Laferton et al., 2018). For TADS, AEs were recorded poorly, so we cannot be confident that we have accurately appraised their influence.

We undertook a secondary analysis without prespecified hypotheses, so our statistical analysis needs to be treated cautiously.

### Implications and future research

Few studies document the degree of unblinding and the impact of expectancy effects on RCTs (Scott et al., 2022). Recent analyses found only between 5% and 7% of antidepressant trials included testing of the integrity of the double blind, and none of these were funded by pharmaceutical companies (Lin et al., 2022; Scott et al., 2022).

Our analysis suggests that the effects that are demonstrated in placebo-controlled trials of antidepressants may represent amplified placebo effects that are a result of the differential distribution of expectancy effects caused by unblinding. Since the expectancy effects are substantial, even a small degree of unblinding might produce an apparent difference between an active drug and a placebo. For future research, there is a clear need for more stringent study designs that systematically record and analyse treatment guesses and assess blindness, and do so early on and repeatedly (Scott et al., 2022). Volunteer studies would also be useful in establishing the degree to which people can identify differences between various antidepressants and placebo.

Our results suggest that future research should routinely assess the occurrence and impact of unblinding, as also suggested by Scott et al (2022). A simple way to do this is to ask participants to guess which treatment they are taking and then factor the effect of guess into the analysis of outcome. Participants should be asked to guess early in the course of the trial, before therapeutic effects would be expected to occur, and then repeatedly thereafter. There may be advantages to avoiding a *don’t know* option; without it, people might reveal suspicions that they would not otherwise volunteer. Further trials using active placebos are also important, especially since no such trial has been conducted with a selective serotonin reuptake inhibitor (SSRI). However, selecting an active placebo that is acceptable and comparable is challenging.

In addition, we urge researchers with access to data about patient or investigator guesses in RCTs, especially where there have been apparently positive outcomes, to analyse and report the extent of unblinding, and the effect of guessing on outcomes to demonstrate how it accords with our findings and what light it sheds on the efficacy of the study drugs.

Many guidelines rely on findings from TADS (e.g. National Institute for Health and Care Excellence (NICE), 2015) in formulating recommendations about the treatment of depression in young people. Antidepressant studies have been criticised for the underreporting of harms (Le Noury et al., 2016) and other methodological problems (Munkholm et al., 2019). If, as our study suggests, antidepressant trials produce inflated estimates of efficacy due to unblinding effects, authors of guidelines might want to review recommendations about the use of antidepressants for children and adults.

## Supplemental Material

sj-docx-1-anp-10.1177_00048674231218623 – Supplemental material for Treatment guesses in the Treatment for Adolescents with Depression Study: Accuracy, unblinding and influence on outcomes Supplemental material, sj-docx-1-anp-10.1177_00048674231218623 for Treatment guesses in the Treatment for Adolescents with Depression Study: Accuracy, unblinding and influence on outcomes by Jon Jureidini, Joanna Moncrieff, Julie Klau, Natalie Aboustate and Melissa Raven in Australian & New Zealand Journal of Psychiatry
