# Supplementary material for: Treatment guesses in the Treatment for Adolescents with Depression Study: Accuracy, unblinding and influence on outcomes
Source: Aust N Z J Psychiatry. 2023 Dec 21;58(4):355–64. doi: 10.1177/00048674231218623 (PMC10960316; doi:10.1177/00048674231218623)
Supplement: sj-docx-1-anp-10.1177_00048674231218623 – Supplemental material for Treatment guesses in the Treatment for Adolescents with Depression Study: Accuracy, unblinding and influence on outcomes [file sj-docx-1-anp-10.1177_00048674231218623.docx]

**Supplementary materials:**

Manuscript: Jureidini, JN, Moncrieff, J, Klau, J, Aboustate, N, Raven, M. Treatment guesses in the Treatment for Adolescents with Depression Study: Accuracy, unblinding, and influence on outcomes. 2023.

Adolescents’ outcome at 6 and 12 weeks according to group allocation and guess are plotted in Figure S1, and further reporting of regression models for adolescent guesses in Tables S2-5.

159 parents guessed their adolescents were allocated to fluoxetine or placebo (pill-only) at week-6. Baseline characteristics for these parents are provided in Table S1 and respective regression models are reported in Tables S6-9.

164 pharmacotherapists guessed adolescents were allocated to fluoxetine or placebo (pill-only) at week-6. Baseline characteristics for these clinicians are provided in Table S5 and respective regression models are reported in Tables S10-13.


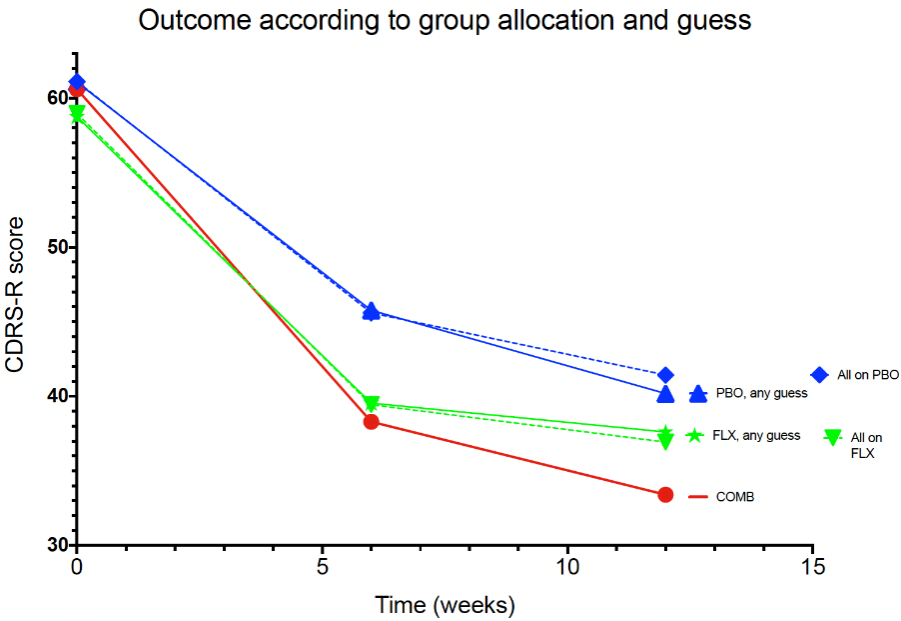


**Figure S1. Change in CDRS-R between week-6 and week-12 for the overall sample cohort allocated to pill-only arms, the cohort of adolescents who had a guess for their allocation at week-6 and the cohort of adolescents whose IEs provided allocation guesses at week-6.**

**Table S1. Mean change (SD) in CDRS-R between baseline and week-12 for adolescents, IEs, parents and pharmacotherapists; including count and t-tests reported with standard mean difference (SMD), and bolded statistically significant p-values (p<0.05).**

| Guessing Group | Count (n) | Mean Change in CDRS-R (SD) | t-test |
| --- | --- | --- | --- |
| Adolescent guessed on FLX | 62 | -26.98 (16.19) | t(139)=-3.96, **p<0.001** (SMD = 0.67) |
| Adolescent guessed not on FLX | 79 | -16.65 (14.73) |  |
| IE guessed on FLX | 101 | -22.92 (15.75) | t(140)=-2.84, **p=0.005** (SMD=0.53) |
| IE guessed not on FLX | 41 | -14.49 (16.63) |  |
| Parent guessed on FLX | 75 | -23.73 (16.65) | t(145)=-2.14, **p=0.034** (SMD=0.35) |
| Parent guessed not on FLX | 72 | -18.07 (15.39) |  |
| Pharmacotherapist guessed on FLX | 84 | -25.06 (15.22) | t(150) = -3.27, **p=0.001** (SMD=0.53) |
| Pharmacotherapist guessed on PBO | 68 | -17.18 (14.23) |  |

**Table S2.** **Model 3 for adolescent cohort: Treatment allocation as a predictor of CDRS-R at week-12, where R^2^=0.088 (n=130, p=0.031) and adjusted R^2^ =0.059.**

| Variable | B | Robust SE | 95% CI | β | t | p-value | ω^2^ |
| --- | --- | --- | --- | --- | --- | --- | --- |
| Treatment allocation | 4.34 | 2.29 | (-0.19,8.87) | 0.160 | 1.90 | 0.060 | 0.019 |
| Treatment expectancy | 4.02 | 2.26 | (-0.46, 8.49) | 0.148 | 1.78 | 0.078 | 0.015 |
| CDRS-R baseline | 0.12 | 0.11 | (-0.07, 0.35) | 0.110 | 1.28 | 0.203 | 0.005 |
| Age at baseline | 1.46 | 0.69 | (0.08, 2.83) | 0.169 | 2.10 | **0.038** | 0.022 |

**Table S3.** **Baseline characteristics of the cohort of adolescents whose IEs guessed fluoxetine or placebo at week-6, where statistically significant p-values (p<0.05) are bolded.**

|  | Total  (n,%) | | Guessed fluoxetine  (n) | Guessed placebo  (n) | p-value |
| --- | --- | --- | --- | --- | --- |
| Adolescent gender |  | 154 (46.8% male) | 41.7% male | 58.7% male | 0.053^$^ |
| Adolescent race |  | 154 (72.7% Caucasian) | 73.2% Caucasian | 71.7% Caucasian | 0.150 |
| Site (n=13) |  | 154 |  |  | 0.231**^%^** |
| Confidence in their guesses at week-6 |  | 153 (32.0% high) | 36.5% high | 21.7% high | 0.74^$^ |
| Adolescent mean (SD) CDRS-R at baseline |  | 154 | 60.3 (10.8) | 58.8 (10.3) | 0.439^§^ |
| Adolescent mean age at baseline |  | 154 | 14.3 (1.5) | 14.6 (1.7) | 0.330^c^ |

^$^Chi square
^%^Analysed using Stata tabchi command, reported Likelihood ratio p-value
^§^t-test, assuming equal variances.

**Table S4.** **Model 1: IE guess at week-6 as predictor of CDRS-R at week-12, where R^2^=0.112 (n=142, p<0.001) and adjusted R^2^ =0.093.**

| Variable | B | Robust SE | 95% CI | β | t | p-value | ω^2^ |
| --- | --- | --- | --- | --- | --- | --- | --- |
| Guess at week 6 | 7.19 | 2.59 | (2.07, 12.31) | 0.237 | 2.78 | **0.006** | 0.051 |
| CDRS-R baseline | 0.12 | 0.11 | (-0.90, 0.34) | 0.095 | 1.14 | 0.254 | 0.003 |
| Age at baseline | 1.57 | 0.68 | (0.23,2.91) | 0.183 | 2.31 | **0.022** | 0.028 |

**Table S5.** **Model 3: Treatment allocation as a predictor of CDRS-R at week-12 in the cohort that had IE guesses for placebo or fluoxetine at week-6, where R^2^=0.131 (n=317, p<0.001) and adjusted R^2^ =0.123.**

| Variable | B | Robust SE | 95% CI | β | t | p-value | ω^2^ |
| --- | --- | --- | --- | --- | --- | --- | --- |
| Treatment arm | 3.82 | 2.25 | (-0.64, 8.28) | 0.277 | 1.70 | 0.092 | 0.013 |
| CDRS-R baseline | 0.10 | 0.11 | (-0.12, 0.32) | 0.007 | 0.88 | 0.380 | 0.001 |
| Age at baseline | 1.77 | 0.66 | (0.46, 3.08) | 0.128 | 2.67 | **0.008** | 0.036 |

**Table S6:** **Baseline characteristics of the cohort of adolescents whose parents guessed fluoxetine or placebo at week-6. Statistically significant p-values (p<0.05) are bolded.**

|  | Total  (n,%) | | Guessed fluoxetine  (%) | Guessed placebo  (%) | p-value |
| --- | --- | --- | --- | --- | --- |
| Adolescent gender |  | 159 (45.9% male) | 50.6% male | 41.3% male | 0.235^$^ |
| Adolescent race |  | 159 (73.6% Caucasian) | 67.1% | 80.0% | 0.340^%^ |
| Site (n=13) |  | 159 |  |  | **0.007**^%^ |
| Treatment expectancy |  | 79 (52.3% much/very much improved) | 60.8% much/very much improved | 44.2% much/very much improved | **0.041**^$^ |
|  |  | 72 (47.7% minimal or no improvement) | 39.2% minimal or no improvement | 55.8% minimal or no improvement |  |
| Confidence in their guesses at week-6 |  | 156 (56.4% high) | 57.1% high | 55.7% high | 0.855^$^ |
| Adolescent mean (SD) CDRS-R at baseline |  | 159 | 60.7 (11.3) | 59.4 (10.4) | 0.435^§^ |
| Adolescent mean (SD) age at baseline |  | 159 | 14.6 (1.5) | 14.4 (1.7) | 0.539^§^ |

^$^Chi square
^%^Analysed using Stata tabchi command, reported Likelihood ratio p-value
^§^t-test, assuming equal variances.

**Table S7.** M**odel 1: Parent guess at week-6 as predictor of CDRS-R at week-12, where R^2^=0.068 (n=139, p=0.083) and adjusted R^2^ =0.040.**

| Variable | B | Robust SE | 95% CI | β | t | p-value | ω^2^ |
| --- | --- | --- | --- | --- | --- | --- | --- |
| Guess at week-6 | 4.91 | 2.35 | (0.26, 9.57) | 0.184 | 2.09 | **0.039** | 0.026 |
| Treatment expectancy | -1.43 | 2.39 | (-6.16, 3.30) | -0.053 | -0.60 | 0.551 | -0.005 |
| CDRS-R baseline | 0.14 | 0.11 | (-0.07, 0.35) | 0.111 | 1.30 | 0.194 | 0.006 |
| Age at baseline | 1.36 | 0.67 | (0.03, 2.69) | 0.165 | 2.03 | 0.045 | 0.020 |

**Table S8.** M**odel 2: Parent guess at week-6 as predictor of CDRS-R at week-12, including treatment expectancy and allocation as predictors; where R^2^=0.069 (n=139, p=0.145) and adjusted R^2^ =0.034.**

| Variable | B | Robust SE | 95% CI | β | t | p-value | ω^2^ |
| --- | --- | --- | --- | --- | --- | --- | --- |
| Guess at week-6 | 4.80 | 2.35 | (0.15, 9.44) | 0.179 | 2.04 | **0.043** | 0.024 |
| Treatment expectancy | -1.50 | 2.43 | (-6.31, 3.30) | -0.056 | -0.62 | 0.536 | -0.004 |
| CDRS-R baseline | 0.14 | 0.11 | (-0.08, 0.35) | 0.109 | 1.27 | 0.206 | 0.005 |
| Age at baseline | 1.36 | 0.67 | (0.04, 2.59) | 0.165 | 2.02 | 0.045 | 0.020 |
| Treatment allocation | 0.75 | 2.27 | (-3.75, 5.24) | 0.028 | 0.33 | 0.743 | -0.007 |

**Table S9.** M**odel 3: Treatment arm as predictor of outcome at week-12, where R^2^=0.039 (n=139, p=0.290) and adjusted R^2^=0.010**.

| Variable | B | Robust SE | 95% CI | β | t | p-value | ω^2^ |
| --- | --- | --- | --- | --- | --- | --- | --- |
| Guess at week-6 | 1.50 | 2.30 | (-3.06,6.05) | 0.056 | 0.65 | 0.517 | -0.004 |
| Treatment expectancy | -0.56 | 2.34 | (-5.18, 4.06) | -0.021 | -0.24 | 0.811 | -0.007 |
| CDRS-R baseline | 0.13 | 0.11 | (-0.09, 0.34) | 0.103 | 1.19 | 0.237 | 0.003 |
| Age at baseline | 1.27 | 0.68 | (-0.07, 2.61) | 0.154 | 1.88 | 0.062 | 0.016 |

**Table S10.** **Baseline characteristics of the cohort of adolescents whose pharmacotherapists guessed fluoxetine or placebo at week-6.** Statistically significant p-values (p<0.05) are bolded.

|  | Total  (n,%) | | Guessed fluoxetine  (%) | Guessed placebo  (%) | p-value |
| --- | --- | --- | --- | --- | --- |
| Adolescent gender |  | 164 (44.5% male) | 46.2% male | 42.5% male | 0.637^$^ |
| Adolescent race |  | 164 (73.2% Caucasian) | 75.8% | 69.9% | 0.103^%^ |
| Site (n=13) |  | 164 |  |  | **0.040**^%^ |
| Confidence in their guesses at week-6 |  | 164 (39.6% high) | 35.2% high | 45.2% high | 0.191^$^ |
| Mean(SD) adolescent CDRS-R at baseline |  | 164 | 59.5 (11.5) | 61.0 (10.6) | 0.408^§^ |
| Mean(SD) adolescent age at baseline |  | 164 | 14.4 (1.5) | 14.6 (1.7) | 0.374^§^ |

^$^Chi square
^%^Analysed using Stata tabchi command, reported Likelihood ratio p-value
^§^t-test, assuming equal variances.

**Table S11. Model 1: Pharmacotherapist treatment guess at week-6 as predictor of adolescent CDRS-R at week-12, where R^2^=0.183 (n=152, p<0.001) and adjusted R^2^=0.166.**

| Variable | B | Robust SE | 95% CI | β | t | p-value | ω^2^ |
| --- | --- | --- | --- | --- | --- | --- | --- |
| Guess at week-6 | 8.49 | 1.98 | (4.58,12.40) | 0.323 | 4.29 | **<0.001** | 0.105 |
| CDRS-R baseline | 0.21 | 0.09 | (0.03, 0.39) | 0.180 | 2.35 | **0.020** | 0.031 |
| Age at baseline | 1.25 | 0.55 | (0.16, 2.34) | 0.155 | 2.26 | **0.025** | 0.021 |

**Table S12. Model 2: Pharmacotherapist guess at week-6 as predictor of adolescent CDRS-R at week-12, including treatment expectancy and allocation as a predictor; where R^2^=0.207 (n=152, p<0.001) and adjusted R^2^ =0.185.**

| Variable | B | Robust SE | 95% CI | β | t | p-value | ω^2^ |
| --- | --- | --- | --- | --- | --- | --- | --- |
| Guess at week-6 | 7.47 | 2.10 | (3.31, 11.63) | 0.284 | 3.55 | **0.001** | 0.080 |
| CDRS-R baseline | 0.20 | 0.09 | (0.02, 0.38) | 0.169 | 2.22 | **0.028** | 0.027 |
| Age at baseline | 1.28 | 0.54 | (0.22, 2.35) | 0.159 | 2.37 | **0.019** | 0.024 |
| Treatment arm | 4.18 | 2.07 | (0.08, 8.28) | 0.160 | 2.01 | 0.046 | 0.022 |

**Table S13. Model 3: Treatment allocation as predictor of outcome at week-12, where R^2^=0.131 (n=152, p<0.001) and adjusted R^2^=0.114.**

| Variable | B | Robust SE | 95% CI | β | t | p-value | ω^2^ |
| --- | --- | --- | --- | --- | --- | --- | --- |
| Treatment arm | 5.97 | 2.00 | (2.02,9.92) | 0.228 | 2.99 | **0.003** | 0.050 |
| CDRS-R baseline | 0.21 | 0.10 | (0.02, 0.40) | 0.177 | 2.20 | **0.029** | 0.028 |
| Age at baseline | 1.50 | 0.56 | (0.40, 2.60) | 0.186 | 2.69 | **0.008** | 0.031 |

**Table S14.** **Distribution of 6-week guesses by adolescents (n, %) according to guess category, and whether they had experienced an AE at any time prior to or at the same time as their guess.** Adolescents guessed they were allocated to fluoxetine more frequently when they experienced an AE before the guess (49% vs 42% without AEs) and guessed placebo more frequently when they did not have an AE before the guess (59% vs 51%). However, cell numbers were too small for statistical testing to be meaningful.

| Adolescent guess | No AE before guess | AE before guess | Total number of adolescent guesses |
| --- | --- | --- | --- |
| Fluoxetine | 39 (42%) | 28 (49%) | 67 (44%) |
| Placebo | 55 (59%) | 29 (51%) | 84 (56%) |
| Total number of guesses | 94 | 57 | 151 |

**Table S15.** **Accuracy of adolescents’ 6-week guesses (n, %), according to whether they had experienced an AE at any time prior to or at the same time as their guess.** Adolescents allocated to fluoxetine guessed correctly more frequently if they had experienced an AE before their guess (71% vs 59% correct guesses without AEs). Adolescents allocated to placebo had similar proportions of correct guesses irrespective of AEs (62% versus 58% without AEs). Cell numbers were too small for statistical testing to be meaningful.

|  | No AE before guess | AE before guess | Total guesses |
| --- | --- | --- | --- |
| Adolescent guessed FLX |  |  |  |
| Incorrect | 16 (41%) | 8 (29%) | 24 (36%) |
| Correct | 23 (59%) | 20 (71%) | 43 (64%) |
| Total number of FLX guesses | 39 | 28 | 67 |
| Adolescent guessed PBO |  |  |  |
| Incorrect | 23 (42%) | 11 (38%) | 34 (41%) |
| Correct | 32 (58%) | 18 (62%) | 50 (60%) |
| Total number of PBO guesses | 55 | 29 | 84 |

**Table S16.** **Distribution of 6-week guesses by IEs (n, %) according to guess category and whether the adolescent had experienced an AE at any time prior to or at the same time as the IE’s guess.** IEs guessed adolescents were allocated to fluoxetine more frequently when the adolescent had experienced an AE before the guess (50% vs 37% without AEs), however cell numbers were too small for statistical testing to be meaningful.

| IE guess | No AE before IE guess | AE before IE guess | Total number of IE guesses |
| --- | --- | --- | --- |
| FLX | 93 (37%) | 50 (50%) | 143 (41%) |
| PBO | 42 (17%) | 20 (20%) | 62 (18%) |
| CBT | 67 (27%) | 13 (13%) | 80 (23%) |
| COMB | 49 (20%) | 18 (18%) | 67 (19%) |
| Total number of guesses | 251 | 101 | 352 |

**Table S17.** **Accuracy of IEs’ 6-week guesses (n, %), according to whether the adolescent had experienced an AE at any time prior to or at the same time as the IE’s guess.** When adolescents were allocated to either fluoxetine or placebo, IEs guessed their allocation correctly more frequently if the adolescent had experienced an AE before their guess (50% with AEs vs 38% without AEs correct for fluoxetine; 60% with AEs vs 38% without AEs correct for placebo). Cell numbers were too small for statistical testing to be meaningful.

|  | No AE before guess | AE before guess | Total guesses |
| --- | --- | --- | --- |
| IE guessed FLX |  |  |  |
| Incorrect | 58 (63%) | 25 (50%) | 83 (58%) |
| Correct | 35 (38%) | 25 (50%) | 60 (42%) |
| Total number of FLX guesses | 93 | 50 | 143 |
| IE guessed placebo |  |  |  |
| Incorrect | 26 (62%) | 8 (40%) | 34 (55%) |
| Correct | 16 (38%) | 12 (60%) | 28 (45%) |
| Total number of PBO guesses | 42 | 20 | 62 |
